# Diagnostic challenges of systemic juvenile idiopathic arthritis with aberrant M-protein expression: a case report

**DOI:** 10.1186/s12887-026-06828-0

**Published:** 2026-04-02

**Authors:** Shanshan Liang, Wenliu Yu, Peiying Zhong, Chuanmin Tao, Chengyao Jia, Li Zhang

**Affiliations:** 1 Department of Laboratory Medicine, West China Hospital, Sichuan University, Chengdu, 610041 China; 2 Department of Clinical Laboratory, Liuzhou Municipal Liutie Central Hospital, Liuzhou, 545000 China; 3 Department of Laboratory, 363 Hospital, Chengdu, 610041 China; 4 Department of hematology, West China hospital, Sichuan University, No. 37 Guoxue Alley, Wuhou district, Chengdu, 610041 China

**Keywords:** Systemic juvenile idiopathic arthritis, Monoclonal protein, Pediatric case, Case report

## Abstract

**Background:**

Systemic juvenile idiopathic arthritis (sJIA), a severe autoinflammatory subtype of JIA, presents diagnostic challenges due to non-specific clinical features and the absence of definitive biomarkers. Current ILAR classification criteria emphasize exclusion of mimicking conditions, yet misdiagnosis rates persist (20%-35%), particularly in early disease stages.

**Case presentation:**

A 15-year-old female presented with persistent high-grade fever, anemia (Hb: 84→64→73→91 g/L), thrombocytosis (PLT 592→1,044 × 10⁹/L), and iron overload. Initial evaluations excluded infections (CMV-positive, treated) but revealed hypocellular marrow (30%) and a polyclonal hypergammaglobulinemia. Naproxen-responsive fever, generalized lymphadenopathy (PET/CT) and evanescent rash suggested autoinflammatory etiology. Hyperinflammation (CRP 265→311 mg/L, ferritin 1,083→1,271 µg/L) and elevated IL-6 (272 ng/L) emerged, with bone marrow ruling out malignancy (initially suspected MGUS based on the presence of M protein bands in serum protein electrophoresis and immunofixation electrophoresis). Final diagnosis aligned with systemic juvenile idiopathic arthritis (sJIA) per ILAR criteria, supported by rash, arthralgia, and IL-6 elevation. Tocilizumab rapidly resolved fever and inflammation. Ongoing multidisciplinary monitoring continues.

**Conclusions:**

This case report highlights a pediatric sJIA patient exhibiting atypical monoclonal (M) protein expression—a finding not typically associated with sJIA—that complicated diagnostic evaluation. It illustrates how hematological abnormalities may complicate immunophenotypic interpretation in JIA, potentially contributing to diagnostic delays.

## Background

Systemic juvenile idiopathic arthritis (sJIA), classified under the International League of Associations for Rheumatology (ILAR) criteria [1], is an autoinflammatory disorder characterized by spiking fever, evanescent rash, polyarticular synovitis, and serositis, frequently accompanied by multiorgan involvement and markedly elevated inflammatory markers. Representing the most severe JIA subtype, sJIA demonstrates a global prevalence of 16.6–40 per 100,000 children, with Asian populations exhibiting higher proportional representation (30%-40% of JIA cases) [2, 3]. Notably, 10%-15% of sJIA patients develop macrophage activation syndrome (MAS)—a life-threatening hyperinflammatory complication associated with mortality rates approaching 50% if untreated [4].

Despite advancements in rheumatological diagnostics, sJIA remains a formidable clinical challenge due to its overlap with other febrile disorders and the absence of disease-specific biomarkers [5]. Current diagnostic frameworks rely heavily on the exclusion of mimics (e.g., infections, malignancies), necessitating meticulous clinical evaluation to mitigate risks of misdiagnosis (estimated at 20%-35% in early disease stages) [2].

This case report describes a pediatric sJIA patient with aberrant monoclonal protein expression, highlighting potential immunological confounders in applying ILAR classification criteria. Our findings underscore the imperative for integrating immunofixation electrophoresis and clonality assays when atypical serological markers emerge during sJIA workup.

## Case presentation

**Fig. 1:**
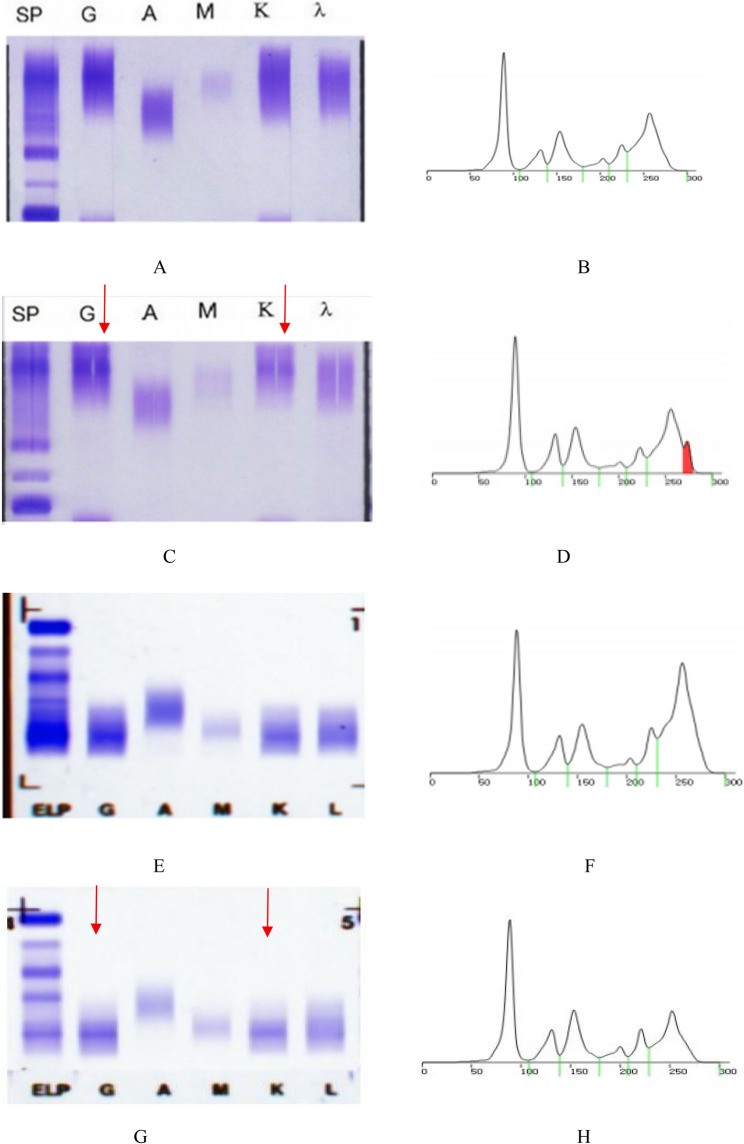
Serial Serum Protein Electrophoresis (SPE, right panels) and Immunofixation Electrophoresis (IFE, left panels) tracking clonal protein status during the disease course. Panels **A** & **B** (October 31, 2023): Initial IFE (**A**) shows no monoclonal (M)-protein bands across IgG (G), IgA (A), IgM (M), κ (K), and λ (λ) fractions; SPE (**B**) displays a broad γ-region peak, consistent with polyclonal hypergammaglobulinemia during early immune activation. Panels **C** & **D** (April 5, 2024): IFE (**C**) identifies a distinct band in the IgG-κ fraction, and SPE (**D**) confirms a discrete M-protein peak in the γ-region. At this stage, clonal dysplasia (e.g., monoclonal gammopathy) was clinically suspected. Panels **E** & **F** (May 23, 2024): IFE (**E**) demonstrates broad-based, diffuse bands across IgG, IgA, IgM, κ, and λ fractions—characteristic of polyclonal immunoglobulin expression, without discrete or sharp monoclonal (M)-protein bands. Corresponding SPE (**F**) shows a widened, indistinct γ-region peak, consistent with polyclonal hypergammaglobulinemia. Panels **G** & **H** (July 17, 2024): IFE (**G**) shows a distinct band in the IgG-κ fraction, while SPE (**H**) displays a corresponding discrete peak in the γ-region

A 15-year-old girl presented to our hematology clinic on October 23, 2023, with a persistent one-month history of low-grade fever. Initial laboratory studies (October 19) revealed significant anemia (hemoglobin 84 g/L) and thrombocytosis (platelets 592 × 10^9^/L). Diagnostic workup demonstrated three key findings: (1) iron overload (extracellular iron 3+), (2) bone marrow hypocellularity (30% cellularity with granulocytic predominance), and (3) negative testing for Epstein-Barr virus and tuberculosis. Serum protein electrophoresis (SPE) and immunofixation electrophoresis (IFE) were performed to evaluate for plasma cell disorders (Fig. 1A & B), showing no monoclonal protein.

### Clinical pivot 1

Due to persistent fever despite initial negative infectious workup, we pursued cytomegalovirus (CMV) testing on November 25, 2023. Results were positive for both IgM and IgG antibodies, prompting referral to infectious disease. She received antiviral therapy until April 2024, yet continued to experience intermittent fevers.

### Clinical pivot 2

**Fig. 2:**
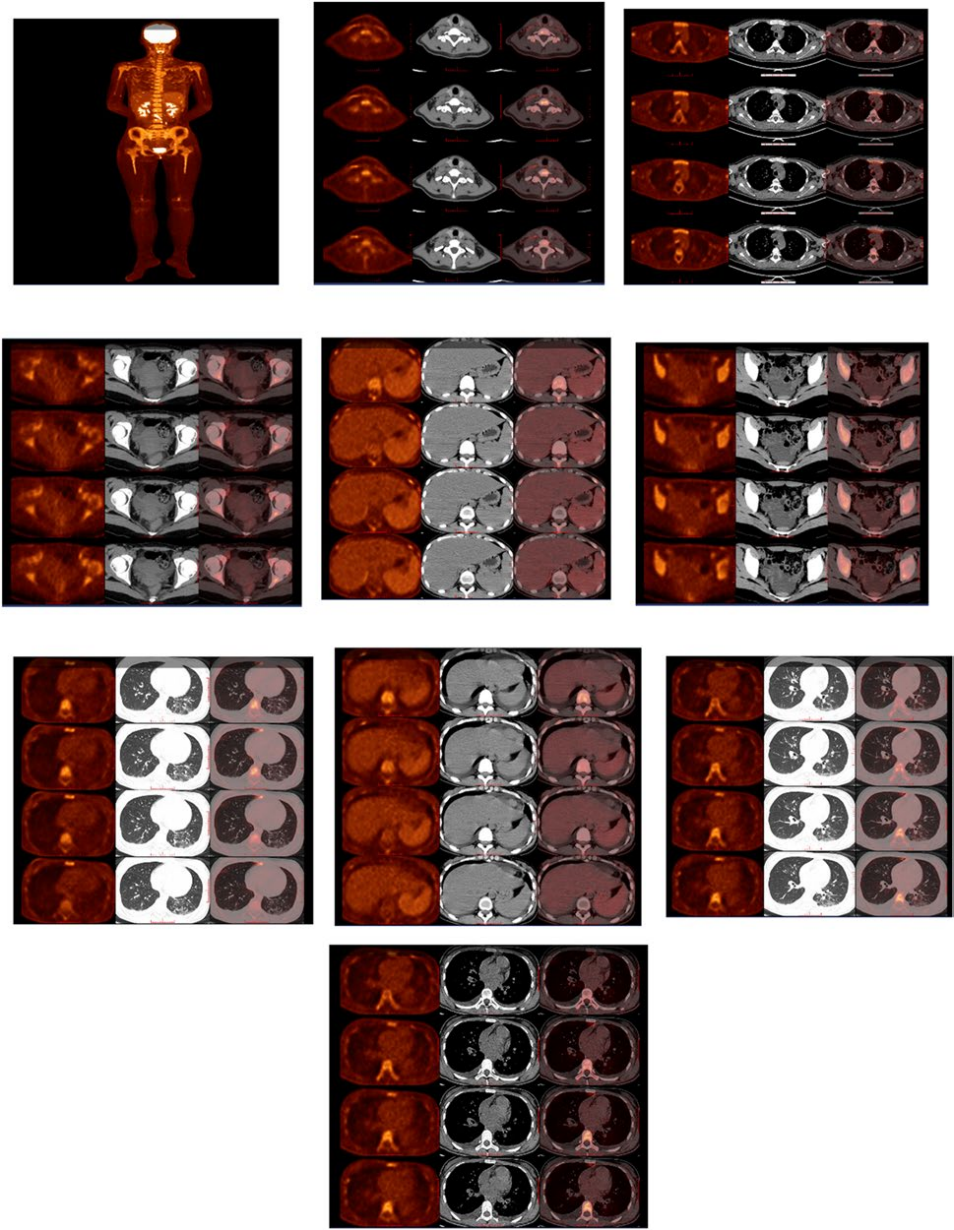
Whole-Body PET-CT Imaging (April 9, 2024): (1) Mildly increased glucose metabolism in cervical/thoracic/abdominal lymph nodes, most likely inflammatory/reactive (clinical correlation and follow-up recommended). (2) Bilateral pulmonary inflammatory changes; ground-glass opacity nodule in right lower lobe (likely inflammatory); bilateral pleural effusions; pericardial effusion. (3) Splenomegaly with mildly increased glucose metabolism; hypermetabolic axial skeleton, suggestive of reactive changes. (4) Hepatomegaly; bilateral adnexal cysts; ascites

On April 2, 2024, she presented to Emergency Department with recurrent high-grade fever (38–40 °C for 10 days). Evaluation revealed fever of unknown origin, pulmonary infection, severe microcytic anemia (hemoglobin 64 g/L, MCV: 77.0 fL), and hypoalbuminemia. She received empiric antimicrobials (meropenem) and supportive care. Admission vitals showed tachycardia (140 bpm) and hyperpyrexia (40.1 °C). Laboratory findings included persistent thrombocytosis (platelets: 598 × 10^9^/L), leukocytosis (WBC: 14.64 × 10^9^/L), profound hypoalbuminemia (28 g/L), and elevated polyclonal immunoglobulins (κ: 34.30 g/L, λ: 13.30 g/L). Autoimmune serologies (ANA, dsDNA, ENA) were negative. Repeat SPE/IFE (Figs. 1C & D) confirmed polyclonal hypergammaglobulinemia. PET/CT demonstrated generalized lymphadenopathy (Fig. 2).

### Critical observation

During hospitalization, antibiotics were escalated for suspected polymicrobial infection. Remarkably, her fever resolved completely with naproxen monotherapy – a pattern highly suggestive of autoinflammatory disease. Serial labs showed progressive thrombocytosis (peaking at 1,044 × 10^9^/L) with stable anemia. She was discharged on April 12 with diagnoses of polyclonal hypergammaglobulinemia, anemia of chronic disease, reactive thrombocytosis, and lymphadenopathy.

### Clinical pivot 3

**Fig. 3:**
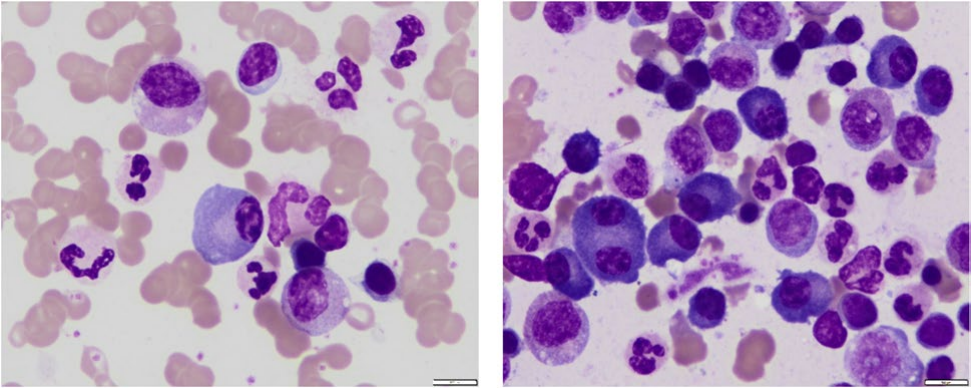
Bone Marrow Smear Examination (May 29, 2024): Bone marrow plasma cells accounted for 11.5%. Rouleaux formation of mature erythrocytes was observed

**Fig. 4:**
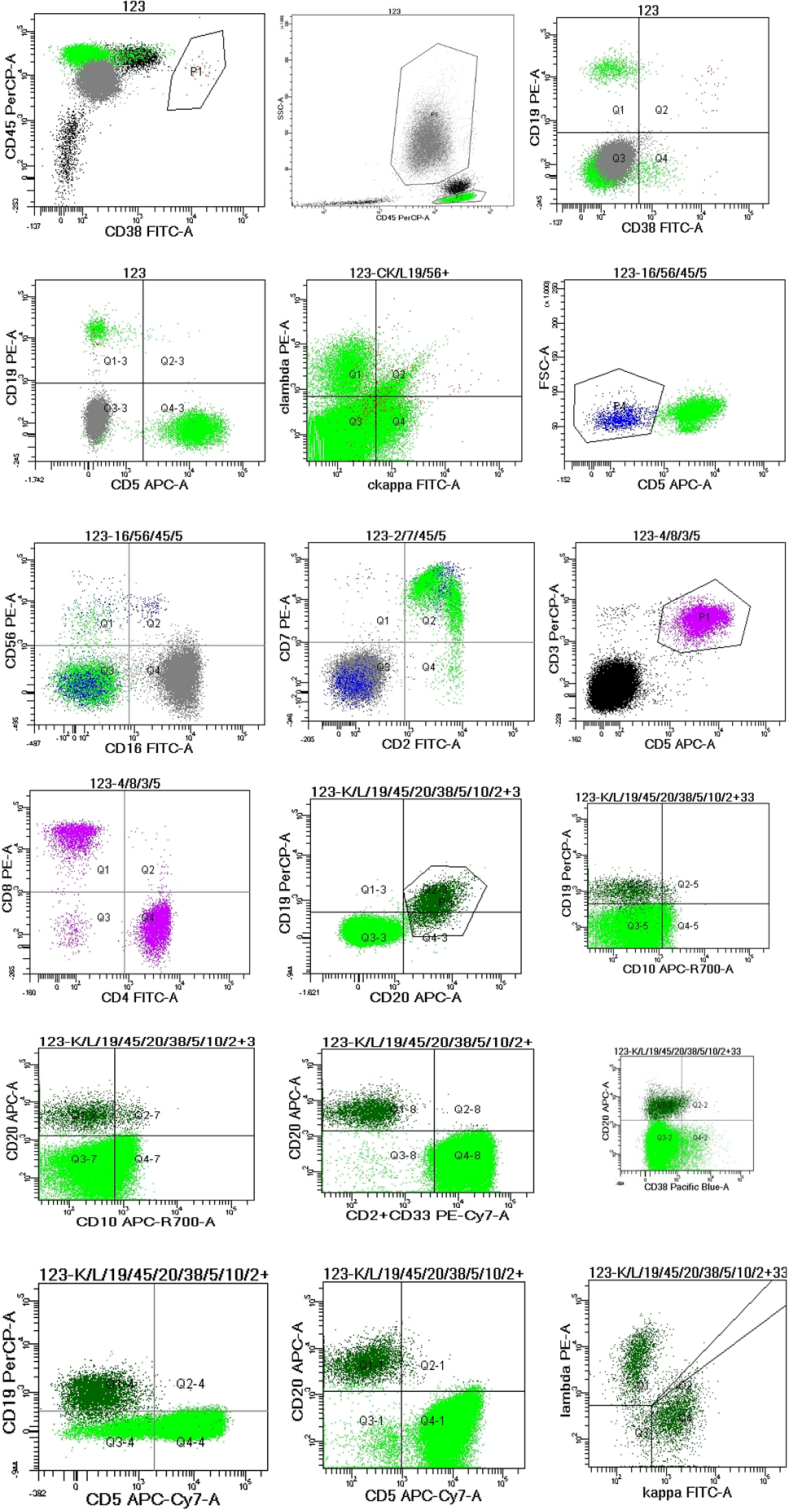
Plasma Cell Immunophenotyping (June 6, 2024): Flow cytometry (FCM) analysis demonstrated no significant aberrant clonal plasma cells

**Fig. 5:**
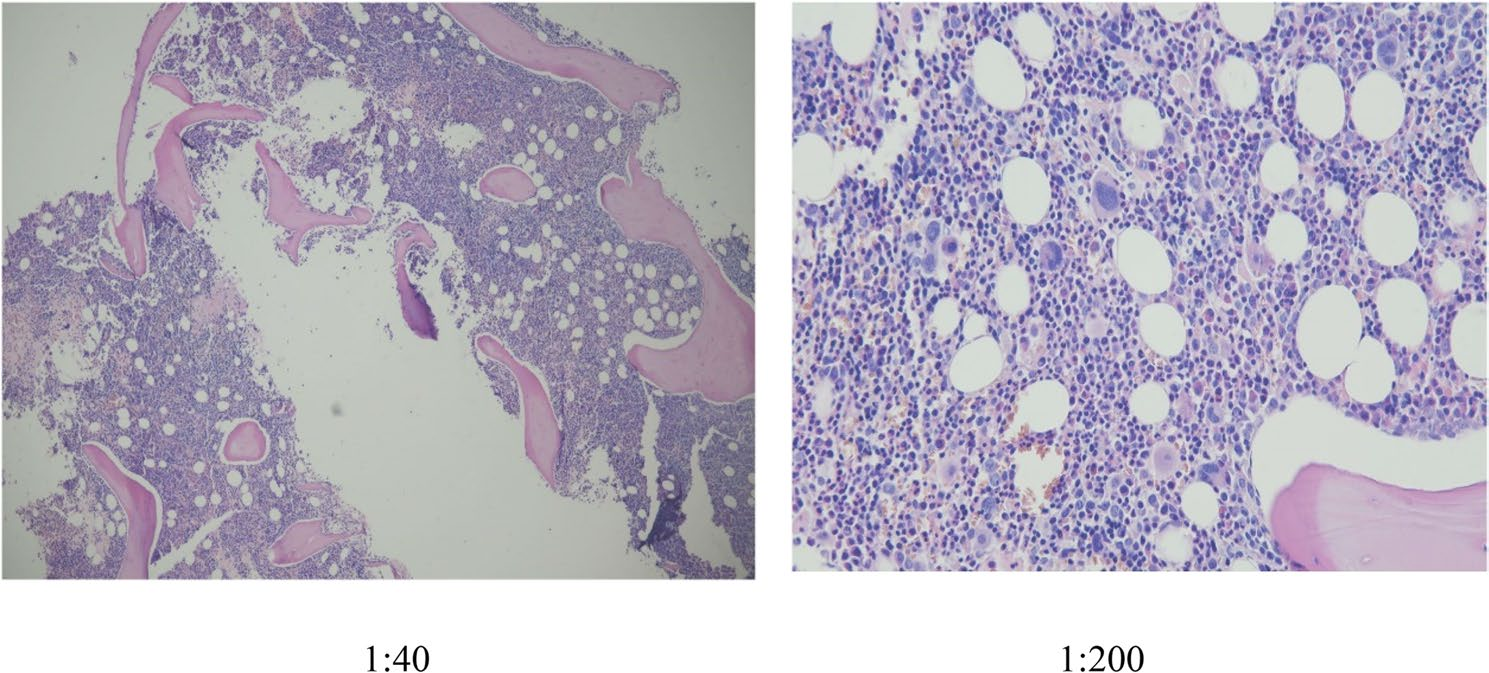
Bone Marrow Biopsy (May 30, 2024): Scant cortical bone fragments. Hematopoietic to adipose tissue ratio approximately 1–2:1. Myeloid to erythroid (M: E) ratio 6–8:1 with granulocytic predominance (MPO-positive). Megakaryocytes 7–16/HPF, occasional small forms identified. Scattered lymphocytes and plasma cells with small focal aggregates CRP

On May 13, 2024, she presented to rheumatology with recurrent morning fever spikes (39.7 °C), productive cough, and profound fatigue. Initial treatment with prednisone and antibiotics failed. Emergency admission revealed a hyperinflammatory state (CRP: 265 mg/L, ferritin: 1,083 µg/L). Repeat bone marrow examination (Figs. 3 and 5) and SPE/IFE (Figs. 1E & F) were performed. Glucocorticoid escalation (hydrocortisone → methylprednisolone → dexamethasone) failed to control fever. Hematology consultation, supported by negative findings on flow cytometry (Fig. 4), bone marrow biopsy (Fig. 5) and normal free light chain κ and λ ratio(1.272, ref 0.31–1.56), excluded hematologic malignancies. This led to a provisional diagnosis of monoclonal gammopathy of undetermined significance (MGUS).

### Therapeutic breakthrough

On June 15, IL-6 receptor blockade with tocilizumab (400 mg IV) achieved complete fever resolution within 24 h. Consolidation therapy with cyclophosphamide (0.8 g IV) was administered on June 17. She was discharged afebrile with normalized inflammatory markers under joint rheumatology/hematology surveillance.

### Definitive diagnosis

Readmission on July 15, 2024 followed two febrile episodes(peak 38.5 °C). Critical historical review revealed childhood-onset recurrent erythematous rash (specific age at onset was not documented in clinical records due to the retrospective nature of the history collection) and transient arthralgias. Laboratory studies showed extreme inflammation (CRP: 311 mg/L, IL-6: 272ng/L, ferritin: 1,271 µg/L) and hematologic dysregulation. Multidisciplinary review confirmed the patient fully met the 2001 International League of Associations for Rheumatology (ILAR) classification criteria for sJIA [1], which require: (1) arthritis (arthritis/arthralgia in ≥ 1 joint, with onset before 16 years of age, lasting ≥ 6 weeks); (2) daily spiking fever (≥ 39 °C) for ≥ 2 weeks; and (3) at least one of the following: evanescent rash, generalized lymphadenopathy, hepatomegaly/splenomegaly, or serositis. This patient aligned with all three core criteria: (1) Arthritis component: Transient arthralgias, documented in longitudinal clinical follow-up, were consistent with the ILAR definition of arthritis-related symptoms in pediatric populations [1]; (2) Fever component: Recurrent quotidian spiking fever (> 39 °C) lasting over 2 weeks (first noted in April 2024, persisted through July 2024), meeting the fever duration and intensity requirement; (3) Additional symptom: Childhood-onset recurrent evanescent rash (historical review) and generalized lymphadenopathy (PET/CT, April 2024, Case Presentation), fulfilling the “at least one additional symptom” criterion.

**Fig. 6:**
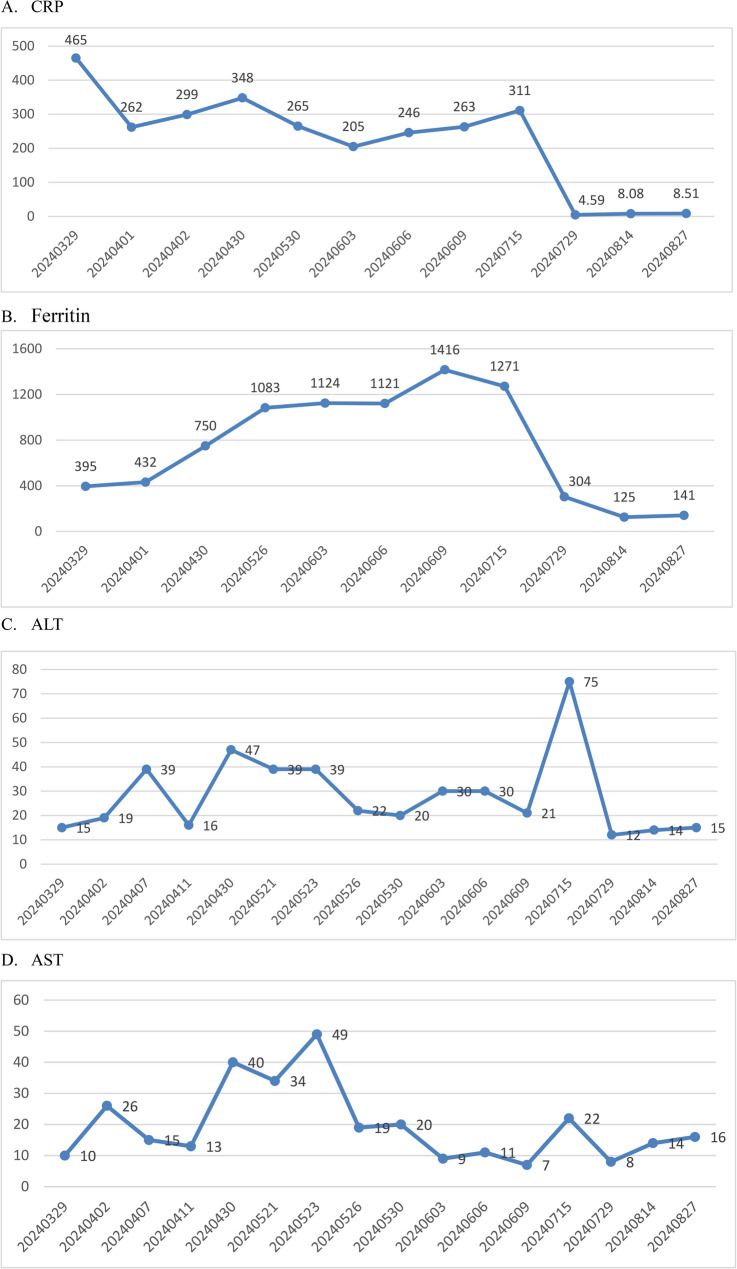
Trends in CRP (**A**), Ferritin (**B**), ALT(**C**) and AST(**D**) levels during hospitalization

Laboratory findings further reinforced the sJIA diagnosis, consistent with core sJIA literature. Marked IL-6 elevation (272ng/L) is a hallmark of sJIA’s hyperinflammatory state, as noted in Reference from Bruck et al. [2], which links IL-6 overproduction to sJIA’s systemic manifestations (e.g., fever, rash) and confirms its role as a key laboratory correlate of ILAR criteria; Hyperferritinemia (1,271 µg/L), while not an independent ILAR criterion, serves as a critical severity marker for sJIA [6] and helped distinguish sJIA from mimics (e.g., infections with lower ferritin levels, malignancies with non-inflammatory ferritin changes), supporting the exclusion of alternative diagnoses. Liver enzymes (ALT and AST) were moderately elevated (Fig. 6C and D), consistent with sJIA-related hepatocellular inflammation—ALT and AST are the key liver enzymes for assessing sJIA-associated liver involvement, as they directly reflect hepatocellular injury [5]. Collectively, the alignment with ILAR criteria and consistency of laboratory findings with sJIA-specific literature confirmed the definitive diagnosis of sJIA.

### Final management

She received IL-6 targeted therapy (tocilizumab 400 mg IV) with methylprednisolone taper on July 17, 2024, and was discharged the following day on biweekly monitoring for maintenance therapy.

### Discussion and conclusions

Current evidence implicates dysregulation of the innate immune system in the pathogenesis of sJIA. The differential diagnosis for sJIA encompasses a broad spectrum, including infectious/post-infectious etiologies, connective tissue disorders, vasculitides, malignancies, and autoinflammatory syndromes [6]. Clinicians should maintain high suspicion for sJIA when encountering pediatric patients with unexplained spiking fever (> 39 °C) accompanied by arthralgia or profound fatigue in the absence of identifiable infectious foci. Notably, serial ferritin monitoring is strongly recommended during diagnostic workup, as hyperferritinemia serves as both a diagnostic biomarker and severity indicator in sJIA.

The diagnosis of sJIA is inherently a prolonged, exclusionary process—given its lack of disease-specific laboratory markers—and laboratory investigations serve two key roles: assessing inflammation severity and ruling out alternative causes, both critical to resolving this case’s diagnostic uncertainty. The patient’s laboratory features aligned closely with classic sJIA signatures in existing literature: her marked elevations in CRP (Fig. 6A), ferritin (Fig. 6B), and IL-6 reflect Murray et al. [3]’s identification of these as markers of sJIA activity/severity, while Bruck et al. [2] highlight IL-6’s role in driving sJIA’s systemic manifestations. Her mild-to-moderate hypochromic anemia, leukocytosis, and thrombocytosis also match Lee et al. [5]’s finding that such inflammation-related hematologic changes occur in 60–70% of active pediatric sJIA cases. These features further distinguished sJIA from hematologic malignancy: Rajkumar et al. [7] note clonal disorders (e.g., MGUS) typically present with cytopenias, unlike the patient’s reactive leukocytosis/thrombocytosis. Paired with negative bone marrow biopsy, flow cytometry, and free light chain results, this excluded malignancy. Ultimately, these laboratory observations—linked to sJIA literature—reinforced the diagnosis while ruling out confounding etiologies. Although targeted genetic testing for monogenic autoinflammatory syndromes was not performed in this case, the classic clinical phenotype, profound elevation of IL-6, and the rapid, complete response to IL-6 receptor blockade provided strong cumulative evidence supporting the diagnosis of sJIA over other monogenic autoinflammatory diseases.

The dynamic changes in SPE and IFE results, as illustrated in Fig. 1, underscore the diagnostic complexities encountered in this case of sJIA and highlight critical considerations for interpreting serological markers in the context of autoinflammatory disease and biologic therapy.

Initially, on October 31, 2023 (Panels A & B), the absence of discrete monoclonal (M)-protein bands on IFE and the broad γ-region peak on SPE were consistent with polyclonal hypergammaglobulinemia—a hallmark of early immune activation in systemic inflammation. This finding aligned with the patient’s initial presentation of persistent fever and hematologic abnormalities, supporting a reactive inflammatory process rather than clonal dysplasia.

By April 5, 2024 (Panels C & D), the emergence of a distinct IgG-κ band on IFE and a discrete γ-region peak on SPE raised legitimate concern for clonal disorders such as MGUS or early plasma cell malignancy. This transient “clonal signal” underscores a key diagnostic pitfall in sJIA: the overlap between hematologic manifestations of severe autoinflammation (e.g., reactive plasma cell expansion) and lymphoproliferative disease markers. Such findings necessitate rigorous exclusion of malignancy through correlative studies, including bone marrow examination and immunophenotyping, as performed here.

Monoclonal gammopathy is characterized by the detection of clonally produced immunoglobulin components - either a monoclonal protein or free light chains (FLC) - in plasma, urine, or both bodily fluids, originating from aberrant proliferation of plasma cells or B lymphocytes. This hematologic abnormality serves as a biomarker for lymphoproliferative disorders including multiple myeloma (MM), immunoglobulin light-chain (AL) amyloidosis, and related malignancies. In atypical clinical scenarios, monoclonal protein deposition may induce tissue injury without concomitant hematologic malignancy, a condition designated as MGCS [7].

While monoclonal gammopathy typically prompts consideration of lymphoproliferative disorders [8], comprehensive evaluation excluded malignancy: bone marrow aspiration revealed 11.5% plasma cells with reactive morphology; immunophenotyping confirmed no evidence of clonal plasma cells (CD56-/CD19+); and initial findings were dominated by polyclonal hyperglobulinemia, evidenced by κ/λ ratio normalization. Notably, repeat testing on May 23, 2024 (Panels E & F) demonstrated a return to polyclonal patterns: broad, diffuse bands across all immunoglobulin fractions on IFE and a broadened, indistinct γ-peak on SPE. This shift starkly contrasted with the earlier abnormalities and reinforced their reactive nature, as hyperinflammatory states in sJIA characteristically drive polyclonal B-cell activation and immunoglobulin elevation—phenomena distinct from true clonal expansion [2, 5, 6]. This transition also paralleled the clinical trajectory of worsening systemic inflammation, further supporting an autoinflammatory (not autoimmune) etiology over clonal disease.

The July 17, 2024 final evaluation (Panels G & H) revealed a critical interpretive challenge: the reappearance of an IgG-κ band on IFE and a corresponding γ-peak on SPE, ultimately attributed to tocilizumab interference. This finding underscores the imperative to integrate therapeutic drug history with serological results. Specifically, within the context of anti-IL-6 receptor therapy, apparent ‘clonal bands’ require rigorous validation against clinical response, clonality assays, and, when indicated, drug-specific interference testing. As an IgG kappa monoclonal antibody, tocilizumab is a well-established cause of artifactual IFE patterns—a phenomenon documented in our prior study [9] and consistent with package insert warnings. Mechanistically, this occurs because tocilizumab’s intrinsic structure mimics monoclonal proteins during electrophoresis. As demonstrated by Shirouchi et al. [10], IgG-κ biologics (e.g., daratumumab, tocilizumab) consistently produce artifactual bands on standard IFE systems due to co-migration with endogenous M-proteins. HYDRASHIFT assays definitively confirmed these bands as drug-derived by selectively shifting the therapeutic antibody band (*P* < 0.001).

Collectively, these serial electrophoretic findings illustrate the dynamic interplay between systemic inflammation, reactive immune responses, and therapeutic artifacts in sJIA. They reinforce that M-protein detection in this population demands a multidisciplinary approach—synthesizing clinical course, inflammatory marker trends, and treatment history—to avoid misclassification of reactive changes as malignancy. For clinicians managing sJIA patients on IgG-κ biologic agents, heightened vigilance for such electrophoretic artifacts is critical to ensuring accurate diagnosis and appropriate therapy.

To our knowledge, this represents the first report of transient monoclonal protein detection in a sJIA patient. This case illustrates how false-positive M-protein results can introduce diagnostic uncertainty during sJIA evaluation, necessitating rigorous correlation between laboratory findings and clinical context. This observation underscores critical clinical considerations: biologic therapies—notably anti-IL-6 agents—may complicate serological interpretation in rheumatological evaluations. Consequently, IFE findings demand integration of therapeutic drug history, clonality assessment (e.g., flow cytometry), and post-discontinuation retesting. In sJIA diagnostics, prioritization of established disease markers (e.g., IL-6 elevation, hyperferritinemia) over transient M-protein detection provides more reliable clinical correlation.

## Data Availability

No datasets were generated or analysed during the current study.
